# Seroprevalence of Hepatitis B Virus and Hepatitis C Virus in Patients Undergoing Maintenance Hemodialysis

**DOI:** 10.7759/cureus.24794

**Published:** 2022-05-07

**Authors:** Usama Bin Shabbir, Wajih Ul Hassan, Ali Raza, Sadia Hafiz, Hafsa Ansar

**Affiliations:** 1 Internal Medicine, Nishtar Medical University, Multan, PAK; 2 Internal Medicine, Mohtarma Benazir Bhutto Shaheed Medical College, Mirpur Azad Kashmir, PAK

**Keywords:** infection control, hemodialysis, seroconversion, seropositivity, hepatitis c, hepatitis b

## Abstract

Background

Underdeveloped countries suffer a huge burden of infectious diseases, among which hepatitis B virus (HBV) and hepatitis C virus (HCV) are of major concern. Our study sought to determine the seroconversion rate for HBV via hepatitis B antigen (HBsAg) and HCV via anti-hepatitis C virus antibodies (anti-HCV) among patients on maintenance hemodialysis in the dialysis unit of a tertiary care hospital in Pakistan.

Methodology

This study was carried out from October 2020 to January 2022 at the hemodialysis unit and the urology department of a tertiary care hospital in Multan. A total of 172 patients of both genders were screened for HBV and HCV seropositivity. Data analysis was done through IBM SPSS Statistics for Windows, Version 25.0 (Released 2017; IBM Corp., Armonk, New York, United States). Conclusions were drawn accordingly.

Results

At the one-year follow-up, screening revealed that 20 (11.7%) patients were positive for HBsAg and 39 (23%) were positive for anti-HCV. Five patients had dual seropositivity for HBsAg and anti-HCV, while three were positive for both HBsAg and anti-HDV. Four out of 20 patients who screened positive for HBsAg and 11 out of 39 patients screened for anti-HCV had a history of one more blood transfusion. Though the correlation between the duration of hemodialysis and viral markers was significant (p<0.05) for the patients screened positive for anti-HCV, a similar correlation was not observed in the patients with seropositivity for HBsAg.

Conclusion

Our study concludes that seroconversion is statistically significant for HCV infection compared to HBV infection. Tight adherence to standard preventive measures to minimize the transmission of these pathogens can lead to a decline in the incidence of these infections. Screening should be done widely to control and optimally manage these infections.

## Introduction

Underdeveloped countries suffer a huge burden of infectious diseases, among which hepatitis B virus (HBV) and hepatitis C virus (HCV) are of major concern [1,2]. The main transmission route for HBV is sexual contact, drug-injecting equipment, and needle sharing [1]. HCV also spreads via multiple blood transfusions, intravenous (IV) drug abuse, and maintenance hemodialysis apparatus [1]. The prevalence of both infectious agents differs geographically. Both HBV and HCV can lead to hepatitis, liver cirrhosis, chronic carrier state, and hepatocellular carcinoma [2].

In patients with end-stage renal disease (ESRD), survival is only possible with renal replacement, either in the form of maintenance dialysis or renal transplant [3]. Although the widespread facilities of maintenance hemodialysis have improved the survival and overall mortality of patients with ESRD, it also carries a large burden of transmissible diseases [4]. Patients who undergo multiple cycles of hemodialysis are at increased risk of various catheter-associated infections, among which HBV and HCV are of major concern [4]. Johnson et al. demonstrated that the patients on maintenance hemodialysis who get infected by either HBV or HCV are at increased mortality risk compared to the general population [5].

The various infection-spread control protocols, guidelines for handling body fluids, isolation strategies, and disinfectants have been developed to minimize the risk of transmission of blood-borne pathogens in patients with ESRD [6]. Despite these infection-limiting efforts, HCV and HBV persist in hemodialysis units, and many patients get infected while on dialysis [7]. Various literature studies have reported seroconversion positivity rates that range from zero to 42% for HCV and zero to 1.8% for HBV [8,9].

Our study sought to determine the seroconversion rate for HBV via hepatitis B antigen (HBsAg) and for HCV via anti-hepatitis C virus antibodies (anti-HCV) among patients on maintenance hemodialysis in the dialysis unit of a tertiary care hospital in Pakistan.

## Materials and methods

This study was carried out from October 2020 to January 2022 at the hemodialysis unit and the urology department of Nishtar Hospital, located in Multan, Pakistan. Approval for the study was taken from the Institutional Review Board (IRB) committee, Nishtar Medical University, Multan, Pakistan (IRB# N2020-0043-9). A total of 172 patients of both genders were randomly screened for HBV and HCV seropositivity. The screening was done at the initiation of hemodialysis and at intervals of three months for a year. Along with medical chart review, the patients were also interviewed for age, sex, co-morbidities, cause of renal failure, history of dialysis, dialysis from any other source, and history of blood transfusion.

Data collection procedure

After taking relevant history and physical examination, screening against HBsAg and anti-HCV was performed. The standard aseptic technique obtained three to five milliliter (ml) of blood. After clotting, serum was separated via centrifugation. A one-step rapid immuno-chromatographic technique was used to screen against HBV and HCV. Enzyme-linked immunosorbent assay (ELISA) was used for confirmation after positive screening for HBsAg and Anti-HCV. The same methodology was repeated at subsequent screenings in the aforementioned study period. The sensitivity and the specificity of the standard kit used were 90% and 95% respectively. 

Data analysis

Data analysis was done through IBM SPSS Statistics for Windows, Version 25.0 (Released 2017; IBM Corp., Armonk, New York, United States). The data were reported as means ± standard error. Statistical comparisons were made using independent 𝑡-tests, paired 𝑡-tests between groups, and a two-tailed 𝑃 value of < 0.05 was considered statistically significant. Conclusions were drawn accordingly.

## Results

Out of the 172 patients included, 170 completed the study. The sociodemographic details and the clinical baseline investigation values of these patients are given in Table 1.

**Table 1:** Sociodemographic details and the clinical baseline investigation values of the patients undergoing hemolysis (first visit).

Parameters	Values (N=170)
Age, years (mean ± SD)	582.±4.7
Gender (%)	
Male	122 (72)
Female	38 (28)
BMI, kg/m² (mean ± SD)	27.22±2.1
Total blood transfusions (mean ± SD)	6.6±1.4
Duration of hemodialysis (%)	
<1year	141 (83%)
>1year	29 (17%)
Baseline hemoglobin, g/dl (mean ± SD)	9.15±2.6
Baseline serum creatinine, mg/dl (mean ± SD)	12.71±6.83
Baseline serum urea, mg/dl (mean ± SD)	156.35±28.33

The underlying cause of chronic renal failure in descending order was; diabetic nephropathy (42%), glomerulonephritis (29%), hypertensive nephropathy (18%), and others (11%). The screening on the first visit revealed that 16 (9.4%) patients were positive for HBsAg and 21 (12.3%) for anti-HCV. Two of the 16 positive patients for HBsAg were also positive for anti-HDV (Table 2). Patients were also screened at subsequent visits. At the one-year follow-up, screening revealed that 20 (11.7%) patients were positive for HBsAg and 39 (23%) were positive for anti-HCV. Five patients had dual seropositivity for HBsAg and anti-HCV, while three were positive for both HBsAg and anti-HDV (Table 2). Four out of 20 patients who screened positive for HBsAg and 11 out of 39 patients screened for anti-HCV had a history of one more blood transfusion. 

**Table 2:** Prevalence of viral hepatitis serum markers in the patients undergoing maintenance hemodialysis for end-stage renal disease (ESRD). HBsAg: hepatitis B antigen; anti-HCV: anti-hepatitis C virus antibodies; Anti-HDV: anti-hepatitis D virus antibodies

Variables	HBsAg (+)	Anti-HCV (+)	Anti-HDV (+)
Initial visit	16 (9.4%)	21 (12.3%)	2 (1.1%)
Follow-up at 1 year	20 (11.7)	39 (23%)	3 (1.8%)
Sex			
Males	13 (10.6%)	24 (20%)	2 (1.6%)
Females	07 (18%)	15 (40%)	1 (2.6%)
Duration of dialysis			
<1 year	06 (4.2%)	11 (78%)	2 (1.4%)
> 1 year	14 (48%)	28 (74%)	1 (2.6%)

Out of the 170 patients, 141 (83%) had taken hemodialysis for less than one year, and 29 (17%) had taken hemodialysis for more than a year. In the case of 39 patients who screened positive for anti- HCV, 11 had undergone hemodialysis for less than a year (28%), and 28 patients had undergone hemodialysis for more than a year (72%) whereas in the case of 20 HBsAg positive patients, six had undergone hemodialysis for less than a year (30%), and 14 patients underwent hemodialysis for more than a year (70%). Although the correlation between the duration of hemodialysis and viral markers was significant (p<0.05) for the patients screened positive for anti-HCV, a similar correlation was not observed in the patients with seropositivity for HBsAg.

## Discussion

Our study reported that the patients with ESRD on maintenance hemodialysis are at increased risk of blood-borne infections, especially from HBV and HCV. The seroprevalence for hepatitis B and C was 9.4% and 12.3%, respectively, at the start of the study. At follow-up at one year, the seroprevalence increased to 12% for HBV infection and 23% for HCV infection. The duration of the hemodialysis was statistically significant in the patients who screened positive for anti-HCV at the one-year follow-up. 

During the early period of hemodialysis management, both HBV and HCV infections are widespread [3]. With the increasing use of aseptic measures, the development of a vaccine against hepatitis B, and isolation strategies, the incidence of seroprevalence for both infections decreased significantly [3]. However, a noticeable decline in reporting was observed in the infection rate of hepatitis B patients. However, despite all the infection control measures, the risk of hepatitis is concerning in the patients undergoing maintenance hemodialysis. 

In general, the incidence and prevalence of HBV and HCV infection in the patients undergoing maintenance hemodialysis reflect the incidence of both infections in the general population [10]. Our study also showed a similar trend. The seroconversion for the HBV was 12% at a one-year follow-up. A survey conducted by Halle et al. reported the same incidence and prevalence rate [6]. In our study, the seroconversion rate for HCV was 23%, which is significantly higher. In literature, there are many studies that show a similar trend. For example, a similar study mentioned above reported a seroconversion of 12% for HCV infection [6]. In 2007, El Amin and colleagues conducted a similar study and reported a seroconversion rate of 17.1% for HCV infection in the patients undergoing maintenance hemodialysis [11]. 

HBV infection causes both acute and chronic hepatitis. Most of the patients (90%) recover from the acute infection by forming neutralizing antibodies, and the rest of the patients develop chronic hepatitis B [1]. Similarly, HCV infection also manifests as both acute and chronic hepatitis C. Most cases develop a chronic infection that leads to cirrhosis, hepatocellular carcinoma, cardiovascular disorders, and neurological manifestation like hepatic encephalopathy [12,13]. Moreover, hemodialysis patients suffer from anxiety and depression, which can be exacerbated by this infection, leading to increased morbidity and mortality [14]. 

Segregation of the positive patients and their instruments from those at increased risk of infection, proper sterilization of the medical equipment, vaccination against HBV, periodic serological monitoring, careful blood transfusions, effective screening measure, and better education of healthcare staff and the patients can lead to decrease the risk of transmission of both HBV and HCV in patients undergoing maintenance hemodialysis [15]. 

Routine blood screening should be done along with proper education and awareness to help early detection of these cases. If controlled at the initial stages, the patients can be saved from extra disease burden, psychological manifestations of the disease, and drastic physical presentations. In addition, the incidence and prevalence trends can be acquired through the blood bank data to formulate a decisive infection control and management plan. 

Our study has a few limitations. First, the sample size was not adequate to reflect a generalized trend in the patients undergoing hemodialysis. Second, some patients had combined HBV and HCV infection; for them, the individual risk for both infections could not be determined. Further longitudinal studies with a large patient population should be carried out in the future.

## Conclusions

Our study concludes that seroconversion is statistically significant for HCV infection compared to HBV. Tight adherence to standard precautionary measures to minimize the transmission of these pathogens can lead to a decline in the incidence of these infections. Screening should be done widely to control and optimally manage these infections.
